# The First Three Years of Childhood

**Published:** 1886-01

**Authors:** 


THE FIRST THREE YEARS OF CHILDHOOD. By Bernard Perez. Edited and translated by Alice M. Christie, translator of Child and Child Nature, etc. An introduction by James Sully, M. D. Chicago: A. N. Marquis & Co. 1885.

This book treats copiously on the various phases of child-life, abounds with practical hints on the proper training of the very young, and forms a practical guide to the parent and the teacher.

